# Heliozoan cellular architecture predicts prey capture performance and growth rate

**DOI:** 10.64898/2026.10.19.760065

**Published:** 2026-10-23

**Authors:** Paul E Schavemaker, Michael Lynch

## Abstract

Prey capture is essential for growth in many species of single-celled eukaryotes, and its various elaborations phenotypically define major eukaryotic clades. Despite impressive work on the mechanistic basis, capture rates, and trade-offs in prey capture, in flagellates in particular, no quantitative assessment of the full fitness contributions of specific prey capture mechanisms has been performed. Here, a step in the direction of such an assessment is taken for the axopod-based prey capture mechanism of heliozoans. A model was established that (1) obtains prey capture rates from a geometrically accurate simulation of prey capture, (2) assesses the energetic cost of the prey capture mechanism as a function of axopod length and number, and (3) uses the capture rates and cost to calculate a cell division time. The heliozoan prey capture model captures the essence of the mechanism and performs decent predictions of axopod cost, axopod length, axopod number, prey capture rate, clearance rate, and cell division time. The core elements of the capture model are broadly applicable and, when supported by mechanistic information, can be applied to flagellates, ciliates, amoeba and other organisms, forming a basis for elucidating how such varied mechanisms of prey capture were able to evolve.

## Full Text Availability

The license terms selected by the author(s) for this preprint version do not permit archiving in PMC. The full text is available from the preprint server.

